# The second complete mitogenome of *Scolopsis ciliata* (Perciformes, Nemipteridae) to analyze control region structure and intraspecific variation

**DOI:** 10.1080/23802359.2019.1675546

**Published:** 2019-10-11

**Authors:** Ren-Xie Wu, Ben-Ben Miao, Su-Fang Niu, Yun Zhai, Hao-Ran Zhang, Fang Liu, Chun-Xiao Ou

**Affiliations:** a College of Fisheries, Guangdong Ocean University, Zhanjiang, Guangdong, P. R. China;; b School of Life Sciences, East China Normal University, Shanghai, P. R. China;; c Guangdong Leizhou Rare Marine Life National Nature Reserve, Zhanjiang, Guangdong, P. R. China

**Keywords:** *Scolopsis ciliata*, mitogenome, control region, intraspecific variation

## Abstract

In this study, we reported the complete mitogenome of *Scolopsis ciliata* sampled from the Beibu Gulf, South China Sea. It was 16,734 bp in length and contained 37 canonical mitochondrial genes and 2 non-coding regions. Fifty-one single nucleotide polymorphisms and five insertion and deletions were detected between two *S. ciliata* mitogenomes, suggesting a high level of intraspecific genetic variation in the species. The control region contained termination associated sequence domain (TAS), central conserved domain (CSB-F, CSB-E, CSB-D, CSB-C, and CSB-A), and conserved sequence block domain (CSB-1, CSB-2, and CSB-3). The phylogenetic analysis of 21 complete mitogenome sequences well supports the phylogenetic position of *S. ciliata* and reveals the phylogenetic relationship of the genus *Scolopsis*.

The saw-jawed monocle bream *Scolopsis ciliata* (Lacépède 1802) is one of the economically important nemipterid species, which widely distributes in the Indo-West Pacific Ocean, reaching west to the Andaman Sea, east to Solomon Islands and Vanuatu, north to the Ryukyu Islands, and south to Australia (Russell 1990, p.109). It usually occurs on sandy bottoms close to coral reefs and is one of the artisanal and commercial fisheries in the northern South China Sea, Gulf of Thailand, and Indonesia (Liu et al. 2016, p. 208). Recently, the complete mitogenome of *S. ciliata* from the coastal water of Malang, East Java, Indonesia was sequenced by Andriyono et al. (2019). However, the control region structure has not been dissected. In addition, the intraspecific genetic variation of *S. ciliata* is expected to be high due to it has obvious habitat preference and low dispersal capacity (Shao 2018). So, in this study, we reported another complete mitogenome of *S. ciliata* sampled from the Beibu Gulf, which is about 3200 Km from Malang, to analyze the control region structure and reveal the genetic variation of mitogenome sequence.

The *S. ciliata* sample was collected from the Guangdong Leizhou Rare Marine Life National Nature Reserve (20°39′09″N, 109°44′08″E), The Beibu Gulf, South China Sea in May 2015. The sample was preserved in 95% ethanol and deposited in Guangdong Ocean University (No. 20150525073). Genomic DNA sequencing was performed using Illumina Hiseq 2500 System (Illumina Inc., San Diego, CA), and a total of 20,023 sequence reads were generated for the mitogenome of *S. ciliata* with an average depth of 299×. The complete sequence of *S. ciliata* mitogenome (GenBank accession number: MN331658) was 16,734 bp in length with a relatively high A + T content (61.91%). The sequence contained 13 protein-coding genes, 22 tRNA genes, and 2 rRNA genes, and 2 non-coding regions,which were similar to other nemipterids mitogenome (Li et al. 2016; Wu et al. 2016, 2017; Wu and Li 2016; Zhai et al. 2019).

Based on the alignment of two *S. ciliata* mitogenomes (MH995531, MN331658), 51 single nucleotide polymorphisms (SNPs) and 5 insertion and deletions were found. The amount of sequence variations in *S. ciliata* reflects a higher level of genetic diversity in comparison with other nemipterids. This may be caused by the long-term geographic isolation between the Indian and West Pacific oceans (Wu et al. 2018), as the two analyzed samples come from each ocean. Among 51 SNPs, 26 SNP were detected in ten protein-coding genes and *tRNA^Pro^*, while 25 SNPs and all insertion and deletions were detected in control region. This suggests that the control region could be used as a molecular marker for exploring the intraspecific micro-evolutionary events of *S. ciliata.*

The control region of *S. ciliata* could be separated into three domains, the termination associated sequence domain (TAS), the central conserved domain (CCD), and the conserved sequence block domain (CSB). One extended terminal associated sequence (ETAS: TACATATATGTATTATCACCATTACATTATATCAACCA), two repeated TAS motifs (TACAT) and one complementary TAS (cTAS) motif (ATGTA), were recognized in the control region. The CSB-F, CSB-E and CSB-D were easily detected in the CCD of *S. ciliata* due to their highly conserved among fishes (Lee et al. 1995). After that, the CSB-C, CSB-B, and CSB-A could be also identified in turn based on their core sequences GCATAAGTT, ATGGCG and CCATGCC, respectively. In the CSB domain, the CSB1, CSB2, and CSB3 were found at the 3′-end of control region, whose sequences were CATTAAAGCTTTCAAGAGCATAA, TCTAAAAACCCCCCCCTACCCCCCC, and AACCCCCCCGGAAACAGGAA, respectively. The tandem repeat sequences were not detected in 986 bp length of *S. ciliata* control region.

The phylogenetic analysis of the bootstrapped neighbor-joining (NJ) tree was constructed by MEGA6.06 programme (Tamura et al. 2013) based on 21 complete mitogenome sequences of Percoidei fishes. The NJ tree (Figure 1) showed that two individuals of *S. ciliata* firstly gathered with the congener *Scolopsis vosmeri*, then clustered together with *Nemipterus* and formed a monophyly of family Nemipteridae. They constituted a sister-group relationship with other families. The present result well supports that the genus *Scolopsis* belongs to the family Nemipteridae.

**Figure 1.**
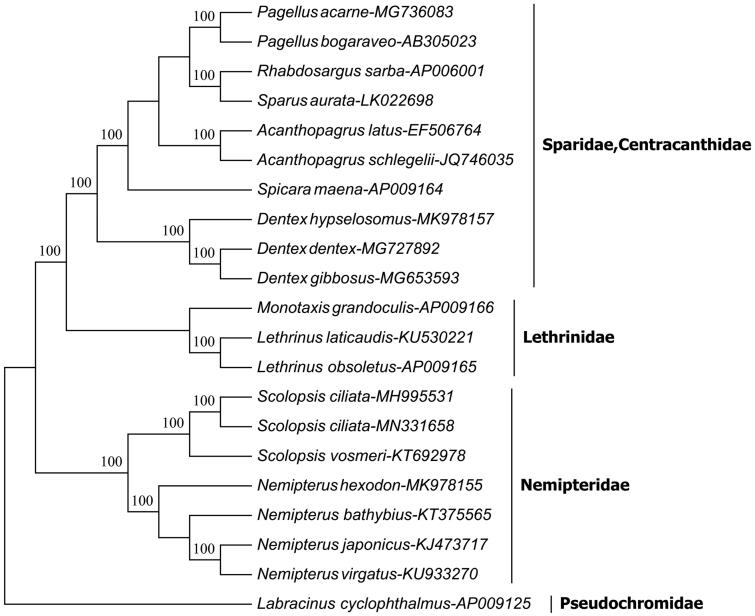
The neighbor-joining phylogenetic tree of 21 complete mitogenome sequences of the Percoidei fishes including two individual of *S. ciliata*. The bootstrap value was given for each branch.
